# Comparative Study of Antemortem Computed Tomography (CT) Findings With Autopsy Findings in Head Injury Cases

**DOI:** 10.7759/cureus.111327

**Published:** 2026-06-22

**Authors:** Toshal D Wankhade, Chaitanya Mittal, Amit Patil, Metta Yaswanth Reddy, Srinivasan D.

**Affiliations:** 1 Forensic Medicine and Toxicology, All India Institute of Medical Sciences, Patna, Patna, IND

**Keywords:** autopsy, computed tomography, forensic radiology, head injury, traumatic brain injury, virtopsy

## Abstract

Introduction: Head injury is a major cause of morbidity and mortality worldwide, and accurate assessment of traumatic head injuries is important for both clinical management and medicolegal investigations. Computed tomography (CT) is routinely used for the evaluation of head injuries in living patients, whereas medicolegal autopsy remains the standard method for postmortem assessment. This prospective comparative observational study was conducted to compare antemortem CT scan findings with autopsy findings in fatal head injury cases.

Methodology: A total of 34 cases that underwent both antemortem CT examination and subsequent medicolegal autopsy were included. Findings related to scalp injuries, skull fractures, extradural hemorrhages (EDHs), subdural hemorrhages, subarachnoid hemorrhage (SAH), brain contusions, brain lacerations, intraventricular hemorrhage (IVH), and cerebellar herniation were compared. Agreement between CT scan and autopsy findings was assessed using percentage agreement, Cohen’s kappa coefficient, and McNemar’s test.

Results: Substantial agreement was observed for skull fractures, EDH, IVH, and cerebellar herniation, whereas lower agreement was observed for scalp injuries, SAH, and brain lacerations.

Conclusions: Antemortem CT scan demonstrated good concordance with autopsy for skull fractures and major intracranial hemorrhages and proved to be a valuable adjunct in the evaluation of fatal head injury cases. However, a CT scan could not completely replace conventional medicolegal autopsy. These findings support the complementary role of CT imaging in forensic practice and its potential contribution to the development of CT-based virtual autopsy in head injury cases. Furthermore, by directly comparing antemortem CT findings with subsequent autopsy findings, this study provides evidence that may facilitate future integration of antemortem CT data into medicolegal practice and guide further research on CT-based virtual autopsy, particularly in resource-limited settings.

## Introduction

Head injury is a trauma resulting in gross or subtle structural changes in the scalp, skull, and/or intracranial contents caused by mechanical forces [1]. Head injury remains a major public health problem and is a significant cause of morbidity and mortality worldwide [2]. In India, traumatic brain injury constitutes a substantial burden of trauma-related morbidity and mortality [3]. Therefore, fatal head injury cases constitute a substantial proportion of the medicolegal autopsy workload.

Accurate identification of head injuries is essential for both clinical management and medicolegal evaluation. In living patients, computed tomography (CT) is the primary imaging modality for the assessment of traumatic head injuries because it rapidly provides detailed information regarding scalp injuries, skull fractures, intracranial hemorrhages, cerebral edema, and other traumatic lesions [4]. The findings obtained from CT imaging play a crucial role in guiding treatment decisions and determining patient outcomes. Similarly, in fatal cases, medicolegal autopsy assesses the nature and extent of head injuries. Through direct examination of the scalp, skull, meninges, brain, and intracranial structures, autopsy helps to establish the cause of death and provides valuable evidence for medicolegal investigations. Traditionally, autopsy has been regarded as the gold standard for the assessment of fatal traumatic head injuries [5,6].

Recent advances in postmortem imaging have led to the development of the concept of virtual autopsy (virtopsy), in which imaging techniques such as CT can be used to examine the body without extensive dissection. Postmortem CT offers several advantages, including its non-invasive nature, rapid image acquisition, a permanent digital record, and greater acceptability among relatives who may object to a conventional autopsy for religious, cultural, or personal reasons. CT imaging has shown particular value in detecting skull fractures, intracranial hemorrhages, and other traumatic lesions associated with head injury. Despite these advantages, postmortem CT cannot yet completely replace conventional autopsy, as certain pathological findings and detailed tissue examinations still require direct visualization and histopathological assessment [7-9]. Therefore, it is important to evaluate the degree of agreement between antemortem CT findings and autopsy findings in fatal head injury cases.

This study was undertaken to compare antemortem CT scan findings with autopsy findings in head injury cases. The results may help determine the strengths and limitations of CT imaging in detecting various head injury lesions and provide evidence regarding its potential role as an adjunct to, or partial substitute for, conventional autopsy in selected cases. However, as the study is confined to head injury findings, its conclusions should not be interpreted as supporting the replacement of the complete medicolegal autopsy by virtual autopsy.

## Materials and methods

This prospective comparative observational study was conducted at the mortuary of the Department of Forensic Medicine and Toxicology in eastern Bihar, India, after obtaining approval from the Institutional Ethics Committee. The study population comprised fatal head injury cases that underwent antemortem CT at the institute at the time of admission and subsequently succumbed during the course of treatment and were subjected to medicolegal autopsy. The history of the cases was obtained from police inquest reports, information provided by relatives, and hospital medical records.

A consecutive sampling method was employed. Cases were included if they had undergone both antemortem CT head examination and subsequent autopsy, with the most recent CT scan performed within 72 hours before death. Cases were excluded if the CT scan had been performed more than 72 hours before death or if any neurosurgical intervention had been carried out after the CT examination and before death. The total study duration was two years, during which the sample was collected over the period of 1.5 years from January 2024 to June 2025, and a total of 34 cases were examined, satisfying the inclusion and exclusion criteria.

After obtaining informed consent from the next of kin, relevant CT scan reports were retrieved from the hospital medical records department. Autopsy findings were collected from postmortem examination reports. During autopsy, the cranial vault was opened using an oscillating saw following the standard autopsy procedure. After removal of the calvarium, the dura and brain were carefully examined according to the departmental autopsy protocol. Only findings about head injuries were considered for comparison. Collected data include various head injury-related variables, including scalp injuries, skull fractures, extradural hemorrhage (EDH), subdural hemorrhage (SDH), subarachnoid hemorrhage (SAH), brain contusions, brain lacerations, cerebellar herniation, and intraventricular hemorrhage (IVH).

Strict confidentiality of patient information was maintained throughout the study, and all data were used solely for research purposes in accordance with institutional ethical guidelines.

Statistical analysis

The collected data were entered into a Microsoft Excel spreadsheet and analyzed using appropriate statistical software. The frequency and percentage of various head injury findings detected on antemortem CT scan and at autopsy were calculated and compared.

For each injury finding, a 2×2 contingency table was constructed by comparing CT scan findings with corresponding autopsy findings. The agreement between the two modalities was assessed by calculating the overall percentage agreement and Cohen's kappa (κ) coefficient. Kappa values were interpreted as slight (<0.20), fair (0.21-0.40), moderate (0.41-0.60), substantial (0.61-0.80), and almost perfect (>0.80) agreement.

McNemar's test was applied to evaluate the statistical significance of differences between paired CT scan and autopsy findings. A *P*-value of less than 0.05 was considered statistically significant.

## Results

A total of 34 fatal head injury cases fulfilling the inclusion and exclusion criteria were included in the study. The distribution of head injury findings identified by antemortem CT and autopsy is presented in Table 1.

**Table 1:** Frequency of head injury findings on CT scan and autopsy (n = 34).

Findings	Autopsy-positive, *n* (%)	CT-positive, *n* (%)
Scalp injury	28 (82.4)	14 (41.2)
Skull vault fracture	27 (79.4)	25 (73.5)
Base of skull fracture	20 (58.8)	14 (41.2)
Extradural hemorrhage	9 (26.5)	7 (20.6)
Subdural hemorrhage	31 (91.2)	25 (73.5)
Subarachnoid hemorrhage	29 (85.3)	16 (47.1)
Brain contusion	20 (58.8)	24 (70.6)
Brain laceration	18 (52.9)	4 (11.8)
Intraventricular hemorrhage	4 (11.8)	4 (11.8)
Cerebellar herniation	6 (17.6)	6 (17.6)

SDH was the most common finding observed at autopsy, being present in 31 (91.2%) cases, followed by SAH in 29 (85.3%) cases, scalp injuries in 28 (82.4%) cases, and skull vault fractures in 27 (79.4%) cases. On CT examination, skull vault fractures and SDH were each identified in 25 (73.5%) cases, while brain contusions were detected in 24 (70.6%) cases. CT scan detected fewer scalp injuries, base of skull fractures, SAHs, and brain lacerations compared with autopsy findings.

Table 2 presents the agreement between CT scan and autopsy findings along with McNemar's analysis. The highest overall agreement was observed for IVH and cerebellar herniation (94.1% each), followed by skull vault fracture and EDH (88.2% each). Moderate agreement was observed for base of skull fracture and SDH (82.4% each), whereas lower agreement was noted for scalp injuries, SAH, and brain lacerations.

**Table 2:** Agreement between CT and autopsy findings with McNemar's test (n = 34). *Statistically significant (*P* < 0.05). TP, true positive; FN, false negative; FP, false positive; TN, true negative; χ², McNemar's chi-square statistic; df, degrees of freedom

Finding	TP	FN	FP	TN	Agreement (%)	χ²	df	*P*-value
Scalp injury	14	14	0	6	58.8	12.07	1	<0.001
Skull vault fracture	24	3	1	6	88.2	0.25	1	0.625
Base of skull fracture	14	6	0	14	82.4	4.17	1	0.031
Extradural hemorrhage	6	3	1	24	88.2	0.25	1	0.625
Subdural hemorrhage	25	6	0	3	82.4	4.17	1	0.031
Subarachnoid hemorrhage	15	14	1	4	55.9	9.60	1	0.001
Brain contusion	16	4	8	6	64.7	0.75	1	0.388
Brain laceration	4	14	0	16	58.8	12.07	1	<0.001
Intraventricular hemorrhage	3	1	1	29	94.1	0.50	1	1.000
Cerebellar herniation	5	1	1	27	94.1	0.50	1	1.000

McNemar's analysis demonstrated statistically significant differences between CT scan and autopsy findings for scalp injuries (*P *< 0.001), base of skull fractures (*P *= 0.031), SDH (*P* = 0.031), SAH (*P *= 0.001), and brain lacerations (*P *< 0.001). No statistically significant differences were observed for skull vault fractures, EDH, brain contusions, IVH, or cerebellar herniation.

Agreement between CT scan and autopsy findings was further assessed using Cohen's kappa coefficient (Table 3). Substantial agreement was observed for skull vault fractures (*κ* = 0.67), base of skull fractures (*κ* = 0.65), EDH (*κ* = 0.66), IVH (*κ* = 0.71), and cerebellar herniation (*κ* = 0.79). Moderate agreement was observed for SDH (*κ* = 0.40), while scalp injuries (*κ* = 0.23) and brain contusions (*κ* = 0.23) showed fair agreement. SAH (*κ* = 0.12) and brain lacerations (*κ* = 0.18) demonstrated only slight agreement between the two modalities.

**Table 3:** Agreement between antemortem CT scan and autopsy findings using Cohen's kappa (κ; n = 34).

Finding	Kappa (κ)	Interpretation
Scalp injury	0.23	Fair agreement
Skull vault fracture	0.67	Substantial agreement
Base of skull fracture	0.65	Substantial agreement
Extradural hemorrhage	0.66	Substantial agreement
Subdural hemorrhage	0.40	Moderate agreement
Subarachnoid hemorrhage	0.12	Slight agreement
Brain contusion	0.23	Fair agreement
Brain laceration	0.18	Slight agreement
Intraventricular hemorrhage	0.71	Substantial agreement
Cerebellar herniation	0.79	Substantial agreement

The diagnostic performance of antemortem CT, considering autopsy as the reference standard, is presented in (Table 4).

**Table 4:** Diagnostic performance of CT compared with autopsy findings (n = 34). PPV, positive predictive value; NPV, negative predictive value

Finding	Sensitivity (%)	Specificity (%)	PPV (%)	NPV (%)	Diagnostic accuracy (%)
Scalp injury	50.0	100.0	100.0	30.0	58.8
Skull vault fracture	88.9	85.7	96.0	66.7	88.2
Base of skull fracture	70.0	100.0	100.0	70.0	82.4
Extradural hemorrhage	66.7	96.0	85.7	88.9	88.2
Subdural hemorrhage	80.6	100.0	100.0	33.3	82.4
Subarachnoid hemorrhage	51.7	80.0	93.8	22.2	55.9
Brain contusion	80.0	42.9	66.7	60.0	64.7
Brain laceration	22.2	100.0	100.0	53.3	58.8
Intraventricular hemorrhage	75.0	96.7	75.0	96.7	94.1
Cerebellar herniation	83.3	96.4	83.3	96.4	94.1

Overall, CT showed good concordance with autopsy and high diagnostic accuracy for the detection of skull fractures and major intracranial hemorrhages. However, autopsy remained superior in detecting scalp injuries, SAH, and brain lacerations.

## Discussion

Frequency of various head injury findings

In the present study, SDH was the most frequently observed intracranial lesion, followed by SAH, scalp injuries, and skull vault fractures.

Various studies have reported different patterns of intracranial hemorrhage in fatal head injury cases. While Wankhade et al. reported SDH as the most common intracranial hemorrhage [10], Alexis et al. and Jayanth et al. reported SAH as the most common [6,11]. Such variations may be attributed to differences in the study population and injury characteristics.

The predominance of intracranial hemorrhages in the present study is consistent with the known pathophysiology of fatal head trauma, in which vascular injury and its associated complications contribute significantly to mortality. In this study, CT scan detected most major intracranial lesions and skull fractures; certain findings, such as scalp injuries, SAH, and brain lacerations, were identified more frequently at autopsy. The various head injury findings are discussed in detail later.

Scalp Injuries

In the present study, scalp injuries were identified in 28 (82.4%) cases at autopsy, whereas the CT scan detected scalp injuries in only 14 (41.2%) cases. The overall agreement between CT and autopsy was 58.8% with fair agreement on Cohen's kappa analysis (*κ* = 0.23), and the difference was statistically significant (*P* < 0.001). These findings indicate that a CT scan has a limited ability to detect external soft tissue injuries of the scalp when compared with direct postmortem examination.

Autopsy allows direct visual inspection and palpation of the scalp, enabling detection of abrasions, contusions, and lacerations that may not be readily appreciable on routine CT imaging. Similar observations were reported by Jayanth et al., who noted that several external injuries identified during autopsy were either under-reported or not documented on CT examination [11]. Likewise, Kuruc et al. also mentioned that CT is less reliable for minor soft tissue injuries, whereas autopsy remains superior for detailed evaluation of soft tissue trauma [12]. These findings suggest that conventional autopsy remains essential for the comprehensive documentation and evaluation of external head injuries. However, the lower detection rate of scalp injuries on CT scan observed in the present study may be partly attributed to the fact that routine CT reports primarily focus on intracranial and skeletal findings, and external soft tissue injuries are often under-reported or not specifically commented upon

Skull Vault Fractures

Skull vault fractures were observed in 27 (79.4%) cases at autopsy and 25 (73.5%) cases on CT scan. The overall agreement between CT scan and autopsy findings for skull vault fractures was 88.2%, with substantial agreement on Cohen's kappa analysis (κ = 0.67). These findings suggest that CT is highly reliable in the detection of skull vault fractures. The excellent visualization of bony structures by CT enables accurate identification of fracture lines, displaced fragments, and comminuted fractures. Similar findings have been reported by Kuruc et al., who found CT to be a useful diagnostic modality for identifying cranial fractures in trauma victims [12]. Singh also observed good concordance between CT scan and autopsy findings for skull fractures, reporting fractures in 77 cases at autopsy and 68 cases on CT scan, with a disparity of only 11.68% [13]. The substantial agreement observed in the present study indicates that CT scan can provide valuable information regarding skull vault fractures and may serve as a useful adjunct to autopsy in fatal head injury cases.

Base of Skull Fractures

Base of skull fractures were identified in 20 (58.8%) cases at autopsy and 14 (41.2%) cases on CT scan. The overall agreement between CT scan and autopsy findings for base of skull fractures was 82.4%, with substantial agreement on Cohen's kappa analysis (*κ* = 0.65). However, autopsy detected additional cases that were missed on CT scan, resulting in a statistically significant difference between the two methods (*P* = 0.031).

The detection of skull base fractures remains challenging because of the complex anatomy of the cranial base and the presence of multiple osseous structures and foramina. Jayanth et al. reported that basal skull fractures were more frequently missed on CT scan than vault fractures, particularly those involving the middle cranial fossa [11]. Similarly, Kuruc et al. noted that CT has limitations in the diagnosis of cranial base fractures despite its good performance in detecting other skeletal injuries [12]. Thus, although CT scan demonstrates good agreement with autopsy for bony injuries and skull fractures in general, its sensitivity for detecting skull base fractures remains comparatively lower.

Extradural Hemorrhage (EDH)

EDH was detected in 9 (26.5%) cases at autopsy and 7 (20.6%) cases on CT scan. The overall agreement between CT scan and autopsy findings for EDH was 88.2%, with substantial agreement on Cohen's kappa analysis (*κ* = 0.66). No statistically significant difference was observed between the two modalities (*P* = 0.625)

Similar studies have reported good CT performance for EDH detection. Panzer et al. observed high specificity for major intracranial hemorrhages, especially EDH on ante-mortem CT [14]. Singh also reported that EDH is well identified on CT, although a few cases may be missed [13].

The substantial agreement observed in the present study indicates that CT scan is a reliable modality for the detection of EDH in fatal head injury cases.

Subdural Hemorrhage

SDH was the most common intracranial lesion in the present study, being observed in 31 (91.2%) cases at autopsy and 25 (73.5%) cases on CT scan. The overall agreement between CT scan and autopsy findings was 82.4%, with moderate agreement on Cohen's kappa analysis (*κ* = 0.40). The difference between the two modalities was statistically significant (*P* = 0.031).

Although CT detected most cases of SDH, some cases identified at autopsy were missed radiologically. Priyatha and Prasannan reported a sensitivity of 50% for SDH detection by CT when compared with autopsy findings, while Das et al. reported a sensitivity of 58% [15,16]. In contrast, Singh observed a relatively small disparity between CT and autopsy findings for SDH, suggesting better CT performance [13]. Similarly, Panzer et al. reported good concordance between antemortem CT and autopsy for major intracranial hemorrhages, including SDH [14]. The present study demonstrated moderate agreement between CT scan and autopsy findings for SDH, which was lower than that observed for EDH.

Subarachnoid Hemorrhage

SAH was identified in 29 (85.3%) cases at autopsy, but only 16 (47.1%) cases on CT scan. The overall agreement between CT scan and autopsy findings for SAH was 55.9%, with slight agreement on Cohen's kappa analysis (*κ* = 0.12). The difference between the two modalities was statistically significant (*P* = 0.001).

SAH was one of the poor-performing injury categories in the present study. Priyatha and Prasannan reported that the sensitivity of CT for detecting SAH ranged from only 8.3% to 22.9% in different regions of the brain [15]. Similarly, Jayanth et al. observed that SAH was the intracranial hemorrhage most frequently missed on CT examination [10]. Das et al. reported a sensitivity of 60.71% for SAH detection on CT scan, indicating that although CT can identify many cases of SAH, a considerable proportion may still be missed when compared with autopsy findings [16]. These findings are consistent with the present study, in which CT failed to detect a substantial number of SAHs identified at autopsy. Thin layers of blood within the subarachnoid space, post-traumatic redistribution of blood, may reduce the sensitivity of CT in detecting SAH. Consequently, autopsy remains superior to CT scan for the identification of SAH.

Brain Contusions

Brain contusions were observed in 20 (58.8%) cases at autopsy and 24 (70.6%) cases on CT scan. The overall agreement between CT scan and autopsy findings for brain contusions was 64.7%, with fair agreement on Cohen's kappa analysis (*κ* = 0.23). No statistically significant difference was observed between the two modalities (*P* = 0.388).

Interestingly, CT identified more brain contusions than autopsy in the present study. Similar observations have been reported by Panzer et al., who demonstrated good specificity of antemortem CT for the detection of cerebral contusions [12]. CT may identify subtle hemorrhagic parenchymal lesions that are difficult to appreciate during routine gross autopsy, or small cortical SAHs that may be interpreted radiologically as cerebral contusions. Furthermore, false-positive CT interpretations, radiological overcalling, and interobserver variability among radiologists and pathologists may have contributed to the observed discordance between CT and autopsy findings.

In contrast, Priyatha and Prasannan reported an overall sensitivity of only 42.9% for hemorrhagic contusions on CT scan when compared with autopsy findings [15]. The variation among studies may be attributed to differences in CT technology, timing of imaging, severity of injury, and autopsy techniques.

Brain Lacerations

Brain lacerations were identified in 18 (52.9%) cases at autopsy, but only 4 (11.8%) cases on CT scan. The overall agreement between CT scan and autopsy findings for brain lacerations was 58.8%, with slight agreement on Cohen's kappa analysis (*κ* = 0.18). The difference between the two modalities was statistically highly significant (*P* < 0.001).

Brain lacerations are frequently under-detected on CT scan when compared with autopsy findings. Das et al. reported extremely low CT sensitivities for cerebral lacerations, ranging from 5.26% for temporal lobe lacerations to 14.29% for frontal lobe lacerations, with most lesions being identified only at autopsy. Similarly, Singh reported complete disparity between CT and autopsy findings for brain lacerations [13,17]. Therefore, direct examination of brain tissue during autopsy remains superior for the detection and characterization of brain lacerations.

Intraventricular Hemorrhage

IVH was observed in four cases on both CT scan and autopsy. The overall agreement between CT scan and autopsy findings for IVH was 94.1%, with substantial agreement on Cohen's kappa analysis (*κ* = 0.71). No statistically significant difference was observed between the two modalities (*P* = 1.000).

Raj et al. observed that IVHs were better demonstrated on CT imaging than on conventional autopsy [8]. The high level of agreement observed in the present study supports the usefulness of CT scans in the identification of IVH in fatal head injury cases.

Cerebellar Herniation

Cerebellar herniation was identified in six cases on both CT scan and autopsy. The overall agreement between CT scan and autopsy findings for cerebellar herniation was 94.1%, with substantial agreement on Cohen's kappa analysis (*κ* = 0.79).

At autopsy, cerebellar herniation is recognized by coning and grooving of the cerebellar tonsils at the foramen magnum, usually in association with cerebral oedema and brainstem compression [18]. The high level of agreement observed in the present study indicates that a CT scan can reliably demonstrate the secondary intracranial changes associated with cerebellar herniation. These findings support the usefulness of CT scans in the identification of cerebellar herniation in fatal head injury cases.

Overall, the findings of the present study demonstrate that antemortem CT scan shows good concordance with autopsy for the detection of skull fractures, EDH, IVH, and cerebellar herniation. However, CT scan was less effective in identifying scalp injuries, SAH, and brain lacerations, for which autopsy remained superior. The observed discrepancies may be attributed to the limitations of CT in detecting subtle soft tissue injuries and certain intracranial lesions, as well as the advantages of direct tissue examination during autopsy.

Limitations of the study

This study was limited by the small sample size and the inclusion of only cases with available antemortem CT scans. Larger multicentric studies are needed to further evaluate the role of CT imaging in fatal head injury cases.

Histopathological examination of brain lesions was not undertaken, and therefore, the comparison was restricted to gross findings observed on CT scan and autopsy.

This study was confined to head injury findings and did not evaluate injuries or pathological changes in other body regions. Therefore, these findings cannot be generalized to the complete medicolegal autopsy procedure.

## Conclusions

Antemortem CT scan demonstrated substantial agreement with autopsy findings for skull fractures, EDH, IVH, and cerebellar herniation, whereas lower agreement was observed for scalp injuries, SAH, and brain lacerations.

CT scan is a valuable adjunct in the evaluation of fatal head injury cases and provides important information regarding bony injuries and major intracranial lesions. However, it cannot completely replace conventional medicolegal autopsy, particularly for the assessment of soft tissue injuries and certain intracranial lesions.

The findings of the present study support the complementary role of CT imaging in forensic practice and its potential contribution to the development of CT-based virtual autopsy in head injury cases.

Furthermore, by directly comparing antemortem CT findings with subsequent autopsy findings, this study provides evidence that may facilitate future integration of antemortem CT data into medicolegal practice and guide further research on CT-based virtual autopsy, particularly in resource-limited settings.
